# Supplementary material for: Revealing the spatial nature of sublattice symmetry
Source: Nat Commun. 2024 May 6;15:3787. doi: 10.1038/s41467-024-48170-y (PMC11074334; doi:10.1038/s41467-024-48170-y)
Supplement: Supplementary file 1 — Supplementary Information [file 41467_2024_48170_MOESM1_ESM.pdf]

Supplementary information for
“Revealing the spatial nature of sublattice symmetry”

Rong Xiao[1] and Y. X. Zhao[2,*]

[1]*National Laboratory of Solid State Microstructures and Department of Physics, Nanjing University, Nanjing 210093, China*
[2]*Department of Physics and HK Institute of Quantum Science & Technology, The University of Hong Kong, Pokfulam Road, Hong Kong, China*

In the Supplementary Information, we provide the derivations of the relations of the band structures under different conventions (Supplementary Note 1), give some detailed information about several lattice models mentioned in the main text (Supplementary Note 2), and demonstrate the boundary effective theory for the topological in-gap edge states (Supplementary Note 3).

Contents

Supplementary Note 1. Relations of band structures under different conventions 1

Supplementary Note 2. Detailed information about lattice models 2

Supplementary Note 3. Boundary effective theory 4

Supplementary References 5

Supplementary Note 1. Relations of band structures under different conventions

In this section, we will derive the relations between the band structures of $\mathcal{H}^{(p)}(\boldsymbol{k})$, $\mathcal{H}^{(d)}(\boldsymbol{k})$, and $h(\boldsymbol{k})$.

Firstly, doubling the unit cell corresponds to folding the BZ in the momentum space [1], in which the points at $k_x/2$ and $k_x/2+\pi$ are mapped to the same point at k_x. Thus, we can obtain the band structures of $\mathcal{H}^{(d)}(\boldsymbol{k})$ by folding that of $\mathcal{H}^{(p)}(\boldsymbol{k})$.

Since the Hamiltonian $\mathcal{H}^{(d)}(\boldsymbol{k})$ can be transformed as Eq. (16) in the Methods, the relation between the band structures of $h(\boldsymbol{k})$ and that of $\mathcal{H}^{(d)}(\boldsymbol{k})$ is given by

$$h:\{\mathcal{E}_n(\boldsymbol{k})\}\mapsto\mathcal{H}^{(d)}:\{|\mathcal{E}_n(\boldsymbol{k})|,-|\mathcal{E}_n(\boldsymbol{k})|\}. \tag{S1}$$

That is, each eigen energy $\mathcal{E}_n(\boldsymbol{k})$ of $h(\boldsymbol{k})$ corresponds to a pair of opposite eigen energies $\pm|\mathcal{E}_n(\boldsymbol{k})|$ of $\mathcal{H}^{(d)}(\boldsymbol{k})$ [2].

Next, we will show that the band structures of $h(\boldsymbol{k})$ are the same as that of $\mathcal{H}^{(p)}(k_x/2,\bar{\boldsymbol{k}})$. Without loss of generality, we consider the case where the primitive unit cell contains an equal number of A and B sublattices, i.e., N is even ($=2M$). In the eigenbasis of $U_S=\sigma_z\otimes 1_M$, $|u\rangle=(|u_A\rangle,|u_B\rangle)^T$, the Hamiltonian in primitive unit cells cannot be off-block diagonalized but still has a generic block structure

$$\mathcal{H}^{(p)}(\boldsymbol{k})=\begin{pmatrix} g(\boldsymbol{k}) & r(\boldsymbol{k})\\ r^\dagger(\boldsymbol{k}) & f(\boldsymbol{k})\end{pmatrix}, \tag{S2}$$

where $g(\boldsymbol{k})$ and $f(\boldsymbol{k})$ are $M\times M$ Hermitian matrices satisfying $g(k_x+\pi,\bar{\boldsymbol{k}})=-g(k_x,\bar{\boldsymbol{k}})$ and $f(k_x+\pi,\bar{\boldsymbol{k}})=-f(k_x,\bar{\boldsymbol{k}})$, while $r(\boldsymbol{k})$ is a $M\times M$ matrix with π-periodicity in k_x, $r(k_x+\pi,\bar{\boldsymbol{k}})=r(k_x,\bar{\boldsymbol{k}})$. Here, $g(\boldsymbol{k})$ and $f(\boldsymbol{k})$ describe the hoppings between two unit cells separated by an odd-length distance along x, while $r(\boldsymbol{k})$ describes the intra-cell hoppings and the hoppings between two unit cells separated by an even-length distance along x. Therefore, the matrices $g(\boldsymbol{k})$ and $f(\boldsymbol{k})$ contain $e^{\pm i(2j+1)k_x}$ terms, while $r(\boldsymbol{k})$ contains $e^{\pm i(2j)k_x}$ terms, with $j\in\mathbb{Z}$.

In doubled unit cells, we label the sublattice A and B in two adjacent primitive unit cells 1 and 2 as $|u_{A_1}\rangle$, $|u_{B_1}\rangle$, $|u_{A_2}\rangle$, and $|u_{B_2}\rangle$, respectively. We choose the special basis $|u\rangle=(|u_{A_1}\rangle,|u_{A_2}\rangle,|u_{B_2}\rangle,|u_{B_1}\rangle)^T$ and the sublattice

*yuxinphy@hku.hk

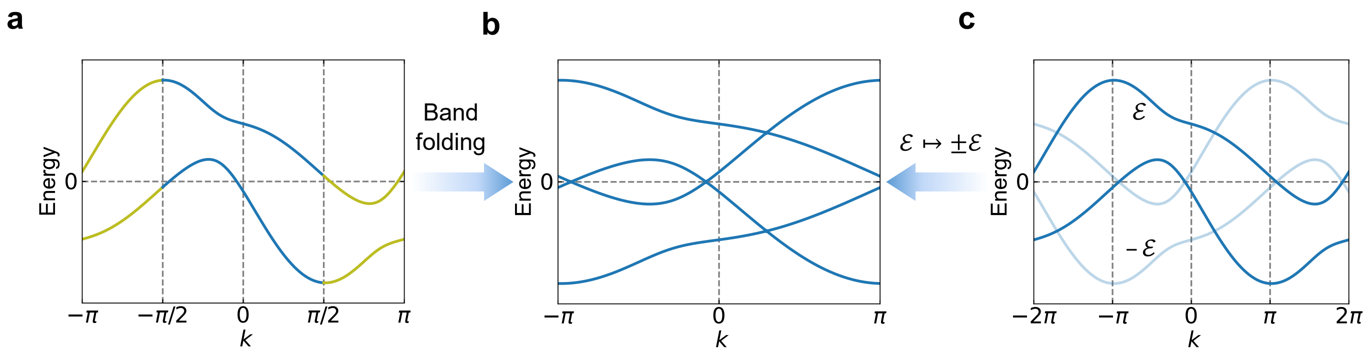

Supplementary Fig. 1: **Relations of band structures.** **a**, **b**, and **c** plot the band structures for $\mathcal{H}^{(p)}(k)$, $\mathcal{H}^{(d)}(k)$, and $h(k)$, respectively.

symmetry is $S = U_S = \tau_z \otimes 1_{2M}$. Then the off-diagonal block of L_x reads

$$R(k_x) = \begin{pmatrix} 1 & 0 \\ 0 & e^{ik_x} \end{pmatrix} \otimes 1_M. \tag{S3}$$

Thus, we have $\det R(k_x) = e^{iMk_x}$. Moreover, doubling the unit cell leads to $k_x \mapsto k_x/2$ but with matrices $g(\boldsymbol{k})$ and $f(\boldsymbol{k})$ associated a factor $e^{\pm ik_x/2}$, namely, the off-diagonal block of the Hamiltonian $\mathcal{H}^{(d)}(\boldsymbol{k})$ becomes

$$Q(\boldsymbol{k}) = \begin{pmatrix} e^{-ik_x/2} g(k_x/2, \bar{\boldsymbol{k}}) & r(k_x/2, \bar{\boldsymbol{k}}) \\ r^\dagger(k_x/2, \bar{\boldsymbol{k}}) & e^{ik_x/2} f(k_x/2, \bar{\boldsymbol{k}}) \end{pmatrix}. \tag{S4}$$

Then the reduced Hamiltonian is

$$h(\boldsymbol{k}) = e^{ik_x/2} R^\dagger(k_x) Q(\boldsymbol{k}) = \begin{pmatrix} g(k_x/2, \bar{\boldsymbol{k}}) & e^{ik_x/2} r(k_x/2, \bar{\boldsymbol{k}}) \\ e^{-ik_x/2} r^\dagger(k_x/2, \bar{\boldsymbol{k}}) & f(k_x/2, \bar{\boldsymbol{k}}) \end{pmatrix}. \tag{S5}$$

It is significant to notice that the reduced Hamiltonian (S5) can be transformed as

$$U' h(\boldsymbol{k}) U'^\dagger = \mathcal{H}^{(p)}(k_x/2, \bar{\boldsymbol{k}}), \tag{S6}$$

where $U'(k_x) = \mathrm{diag}(e^{-ik_x/2}, 1_M)$ and $\mathcal{H}^{(p)}(k_x/2, \bar{\boldsymbol{k}})$ is the Hamiltonian in primitive unit cells by substituting $k_x \mapsto k_x/2$. Therefore, $h(\boldsymbol{k})$ is equivalent to $\mathcal{H}^{(p)}(k_x/2, \bar{\boldsymbol{k}})$ up to a unitary transformation, indicating that their eigen energies are the same.

As shown in Supplementary Fig. 1**a**, **b**, and **c**, we plot the energy spectrum of $\mathcal{H}^{(p)}(k)$, $\mathcal{H}^{(d)}(k)$, and $h(k)$, respectively.

Supplementary Note 2. Detailed information about lattice models

In this section, we will give some detailed information about several lattice models mentioned in the main text.

a. Two 1D lattice models

For the gapless phase, we consider two 1D lattice models, as shown in Fig. 3**a** and **b** of the main text. Each plaquette of the former carries π flux while the latter does not.

In the momentum space, the Bloch Hamiltonian of two models in Fig. 3**a** and **b** of the main text read

$$\mathcal{H}^{(p)}(k) = \begin{pmatrix} 2t\cos(k) & J & J \\ J & -2t\cos(k) & 2\lambda\cos(k) \\ J & 2\lambda\cos(k) & 2t\cos(k) \end{pmatrix}, \tag{S7}$$

and

$$\mathcal{H}^{(p)}(k) = \begin{pmatrix} 2t\cos(k) & J + 2\lambda\cos(2k) \\ J + 2\lambda\cos(2k) & 2t\cos(k) \end{pmatrix}, \tag{S8}$$

respectively. When we set the parameters as $t = J = \lambda = 1.0$, as shown in Fig. 3**c** and **d** of the main text, the band structures of these two models possess six zero modes and eight zero modes, respectively.

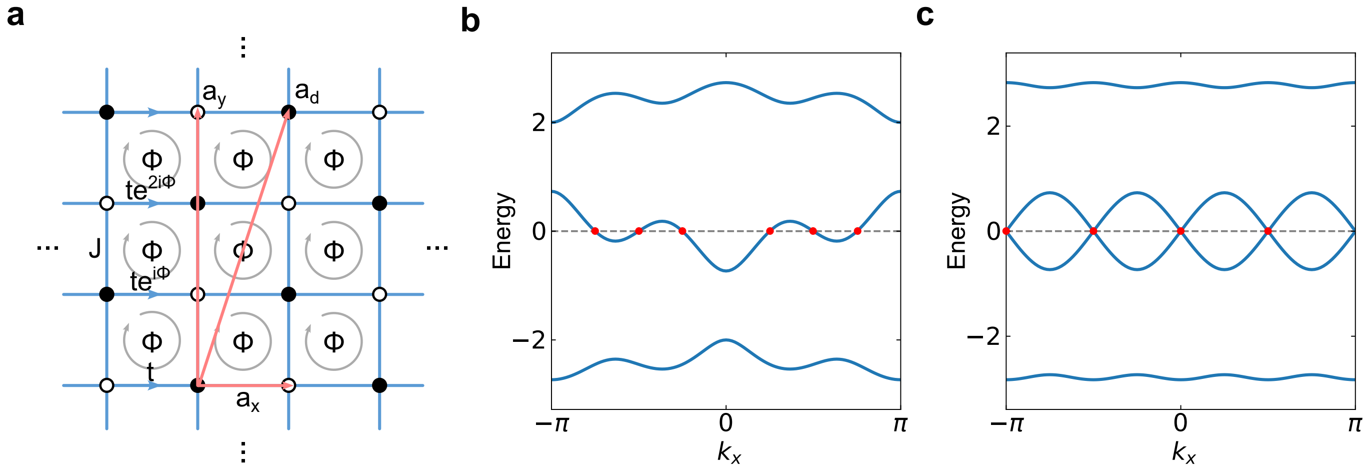

Supplementary Fig. 2: **Zero modes of the Hofstadter model.** **a** Schematic the Hofstadter model with $\Phi = 2\pi/3$. The lattice vectors are indicated by red arrows. t and J stand for the nearest-neighbor hopping amplitudes along x and y directions, respectively. Φ describes the magnetic flux per plaquette. **b** The band structures of the Hofstadter model with $\Phi = 2\pi/3$, $t = J = 1.0$, and $k_d = 0$. **c** The band structures of the Hofstadter model with $\Phi = \pi/2$, $t = J = 1.0$, and $k_y = 0$. There are six zero modes in (b) and eight zero modes in (c).

b. The Hofstadter model

A typical 2D example of the gapless phase is the famous Hofstadter model [3], which describes particles on the 2D square lattice under a uniform magnetic field. We consider that each plaquette of the square lattice carries a rational magnetic flux, $\Phi = 2\pi p/q$, where p and q are coprime integers. The Hofstadter model preserves the sublattice symmetry and is gapless at half filling. There are two magnetic translation operators $L_{x,y}$, which anti-commute with the sublattice symmetry, $\{L_{x,y}, S\} = 0$. Thus, L_y^q anti-commutes with S for odd q, but commutes with S for even q.

When q is odd, we specify the unit cell by $L_x = e^{ik_x}$ and $L_d = L_x L_y^q = e^{ik_d}$ [see Supplementary Fig. 2**a**], so that L_d commutes with S. Then the Bloch Hamiltonian reads

$$\mathcal{H}^{(p)}(\boldsymbol{k}) = \begin{pmatrix} c_0 & Je^{-ik_x} & 0 & \cdots & 0 & Je^{-ik_d} \\ Je^{ik_x} & c_1 & J & \cdots & 0 & 0 \\ 0 & J & c_2 & \cdots & 0 & 0 \\ \vdots & \vdots & \vdots & \ddots & \vdots & \vdots \\ 0 & 0 & 0 & \cdots & c_{N-2} & J \\ Je^{ik_d} & 0 & 0 & \cdots & J & c_{N-1} \end{pmatrix}, \tag{S9}$$

where $c_\alpha = 2t\cos(\alpha\Phi + k_x)$ with $\alpha = 0, 1, \cdots, q-1$. For each 1D subsystem with fixed k_D, $\mathcal{H}^{(p)}(k_x, k_d)$, the center band is partially filled and possesses $4n+2$ zero modes. We plot the band structures of the Hofstadter model with $\Phi = 2\pi/3$, $t = J = 1.0$, and $k_d = 0$ in Supplementary Fig. 2**b**. There are six zero modes in total.

When q is even, the unit cell is specified by $L_x = e^{ik_x}$ and $L_y^q = e^{ik_y}$. Then the Bloch Hamiltonian is

$$\mathcal{H}^{(p)}(\boldsymbol{k}) = \begin{pmatrix} c_0 & J & \cdots & 0 & Je^{-ik_y} \\ J & c_1 & \cdots & 0 & 0 \\ \vdots & \vdots & \ddots & \vdots & \vdots \\ 0 & 0 & \cdots & c_{N-2} & J \\ Je^{ik_y} & 0 & \cdots & J & c_{N-1} \end{pmatrix}, \tag{S10}$$

where $c_\alpha = 2t\cos(\alpha\Phi + k_x)$ with $\alpha = 0, 1, \cdots, q-1$. There is a Dirac semimetal phase at half filling [4], where q Dirac points located at $k_y = 0$ for even $q/2$ but at $k_y = \pi$ for odd $q/2$, indicating $2q = 4n$ zero modes in total. As shown in Supplementary Fig. 2**c**, we plot the band structures of the Hofstadter model with $\Phi = \pi/2$, $t = J = 1.0$, and $k_y = 0$. There are four Dirac points with zero energy, which contributes eight zero modes in total.

c. The dimerized Hofstadter model

Introducing dimerization in the Hofstadter model breaks one primitive magnetic translation and can open a gap at zero energy [5]. The dimerization pattern has a two-site periodicity, resulting in unit cells containing $N = \mathrm{lcm}(2, q)$ lattice sites, where lcm denotes the least common multiple. Therefore, the existence of dimerization preserves the primitive unit cell when q is even, but doubles the unit cell when q is odd. The sublattice symmetry ensures that the Chern number in this half-filling gap is zero. However, there may be novel topological edge states in the half-filling gap, which can be characterized by a $\mathbb{Z}_2$ topological invariant.

We take the dimerized Hofstadter model with $\Phi = \pi/2$ as an example [see Fig. 4**c** of the main text]. The Bloch Hamiltonian of model is

$$\mathcal{H}^{(p)}(\boldsymbol{k}) = \begin{pmatrix} 2t\cos(k_x) & J_1 & 0 & J_2 e^{-ik_y} \\ J_1 & 2t\cos(k_x+\Phi) & J_2 & 0 \\ 0 & J_2 & 2t\cos(k_x+2\Phi) & J_1 \\ J_2 e^{ik_y} & 0 & J_1 & 2t\cos(k_x+3\Phi) \end{pmatrix}. \tag{S11}$$

When we set $t = J_1 = 1.0$ and $J_2 = 2.0$, the path of the Berry phase $\gamma(k_x)$ crosses π once in $k_x \in [-\pi, 0)$, as shown in Fig. 4**b** of the main text. Correspondingly, when we put the system in a slab geometry, there are a pair of in-gap edge states in the half-filling gap [see Fig. 4**d** of the main text].

Supplementary Note 3. Boundary effective theory

The energy dispersion of in-gap edge states in the dimerized Hofstadter model can be exactly obtained by the boundary effective theory. We take the dimerized Hofstadter model with $\Phi = \pi/2$ as an example.

Firstly, the Bloch Hamiltonian (S11) can be written as

$$\mathcal{H}^{(p)}(\boldsymbol{k}) = \mathcal{H}_0(k_x) + \mathcal{H}_1(k_y), \tag{S12}$$

which means that k_x and k_y are separable. We can get the Hamiltonian in real space by applying an inverse Fourier transformation in y direction, namely, replacing the e^{ik_y} (e^{-ik_y}) by the shift operator S_y $(S_y^\dagger)$.

Consider a semi-infinite system $(y > 0)$ with an edge at $y = 0$, then we have $S_y^\dagger|0\rangle = 0$. Taking the ansatz $|\psi\rangle = \sum_{j=0}^{\infty} \rho^j |j\rangle \otimes |\xi\rangle$ with $|\rho| < 1$. Then the Schrödinder equation in the bulk $(j \geq 1)$ is

$$\left[\mathcal{H}_0(k_x) + \mathcal{H}_1(S_y \to \rho, S_y^\dagger \to \rho^{-1})\right]|\xi\rangle = \mathcal{E}|\xi\rangle \tag{S13}$$

and at the boundary $(j = 0)$

$$\left[\mathcal{H}_0(k_x) + \mathcal{H}_1(S_y \to \rho, S_y^\dagger \to 0)\right]|\xi\rangle = \mathcal{E}|\xi\rangle. \tag{S14}$$

The difference between the two equations(S13) and (S14) is the boundary condition

$$T|\xi\rangle = 0, \tag{S15}$$

where

$$T = \begin{pmatrix} 0 & 1 \\ 0_3 & 0 \end{pmatrix} \tag{S16}$$

is called a boundary term and is non-hermitian. Let us conisder two invertible matrices

$$A = \begin{pmatrix} 0_2 & \tau_x \\ \tau_x & 0_2 \end{pmatrix},\ B = \begin{pmatrix} 0_2 & -i\tau_y \\ \tau_x & \tau_0 \end{pmatrix}, \tag{S17}$$

with $A - B = 2T$. Then the boundary condition (S15) becomes

$$(A^{-1}B)|\xi\rangle = |\xi\rangle, \tag{S18}$$

which implies the boundary states is the eigenstates of $(A^{-1}B)$ with the eigenvalue 1. More explicitly, the operator $A^{-1}B = \tau_0 \oplus \tau_z$ has eigenvalues 1, 1, 1, and -1. Then the projector for the edge states on the bottom boundary is given by

$$P^B = \frac{1_4 + (A^{-1}B)}{2} = \begin{pmatrix} 1_3 & 0 \\ 0 & 0 \end{pmatrix}. \tag{S19}$$

The effective Hamiltonian for the edge states on the bottom boundary is

$$\mathcal{H}_{\text{eff}}^{B}(k_x) = P^B \mathcal{H}^{(p)}(\boldsymbol{k}) P^B. \tag{S20}$$

Similarly, we can obtain the projector for the upper boundary

$$P^U = \begin{pmatrix} 0 & 0 \\ 0 & 1_3 \end{pmatrix}. \tag{S21}$$

Then the effective Hamiltonian is

$$\mathcal{H}_{\text{eff}}^{U}(k_x) = P^U \mathcal{H}^{(p)}(\boldsymbol{k}) P^U. \tag{S22}$$

The effective Hamiltonian for a boundary is obtained by simply eliminating all couplings in contact with the opposite boundary. The middle band of $\mathcal{H}_{\text{eff}}^{B,U}(k_x)$ corresponds to the topological edge states in the half-filling gap that are located at the bottom/upper boundary.

Supplementary References

[1] Shao, L. B., Liu, Q., Xiao, R., Yang, S. A. & Zhao, Y. X. Gauge-field extended $k \cdot p$ method and novel topological phases. *Phys. Rev. Lett.* **127**, 076401 (2021).

[2] Tao, T. *Topics in random matrix theory*, vol. 132 (American Mathematical Society, 2023).

[3] Hofstadter, D. R. Energy levels and wave functions of bloch electrons in rational and irrational magnetic fields. *Phys. Rev. B* **14**, 2239–2249 (1976).

[4] Wen, X. & Zee, A. Winding number, family index theorem, and electron hopping in a magnetic field. *Nuclear Physics B* **316**, 641–662 (1989).

[5] Lau, A., Ortix, C. & van den Brink, J. Topological edge states with zero hall conductivity in a dimerized hofstadter model. *Phys. Rev. Lett.* **115**, 216805 (2015).
